# Supplementary material for: All-trans retinoic acid induces synaptic plasticity in human cortical neurons
Source: eLife. 2021 Mar 30;10:e63026. doi: 10.7554/eLife.63026 (PMC8009674; doi:10.7554/eLife.63026)
Supplement: Supplementary file 1. [file elife-63026-supp1.docx]

**Supplementary File 1: Cortical resection samples supplementary information.**

| sample | age [y] | sex | pathology | region | site |
| --- | --- | --- | --- | --- | --- |
| 1 | 47 | m | epilepsy | temporal | right |
| 2 | 59 | m | tumor | frontal | right |
| 3 | 56 | m | tumor | frontal | left |
| 4 | 70 | f | tumor | temporal | left |
| 5* | 55 | m | tumor | temporal | right |
| 6 | 55 | m | tumor | frontal | right |
| 7 | 52 | m | tumor | frontal | left |
| 8** | 58 | m | tumor | temporal | left |
| summary | Ø = 56.5 years | m:7 / f:1 | e:1 / t:7 | temp.:4 / front.:4 | r:4 / l:4 |

*Sample 5 has been used for anisomycin experiments only.

**Sample 8 has been used for immunogold labeling experiments only.
