# Supplementary material for: All-trans retinoic acid induces synaptic plasticity in human cortical neurons
Source: eLife. 2021 Mar 30;10:e63026. doi: 10.7554/eLife.63026 (PMC8009674; doi:10.7554/eLife.63026)
Supplement: Transparent reporting form [file elife-63026-transrepform.pdf]

eLIFE
1st Floor
24 Hills Road
Cambridge CB2 1JP, UK
P 01223 855340
W elifesciences.org
T @elife

eLife's transparent reporting form

We encourage authors to provide detailed information *within their submission* to facilitate the interpretation and replication of experiments. Authors can upload supporting documentation to indicate the use of appropriate reporting guidelines for health-related research (see EQUATOR Network), life science research (see the BioSharing Information Resource), or the ARRIVE guidelines for reporting work involving animal research. Where applicable, authors should refer to any relevant reporting standards documents in this form.

If you have any questions, please consult our Journal Policies and/or contact us: editorial@elifesciences.org.

Sample-size estimation

- You should state whether an appropriate sample size was computed when the study was being designed
- You should state the statistical method of sample size computation and any required assumptions
- If no explicit power analysis was used, you should describe how you decided what sample (replicate) size (number) to use

Please outline where this information can be found within the submission (e.g., sections or figure legends), or explain why this information doesn't apply to your submission:

Sample size estimation was determined in the ethics committee approval AZ593/19 (p.19, l.4-7):

Type I error: 1.6%
Estimated Effect Size prior to experimental assessment: ~1.3 across experiments
Statistical Power: 90%

Replicates

- You should report how often each experiment was performed
- You should include a definition of biological versus technical replication
- The data obtained should be provided and sufficient information should be provided to indicate the number of independent biological and/or technical replicates
- If you encountered any outliers, you should describe how these were handled
- Criteria for exclusion/inclusion of data should be clearly stated
- High-throughput sequence data should be uploaded before submission, with a private link for reviewers provided (these are available from both GEO and ArrayExpress)

Please outline where this information can be found within the submission (e.g., sections or figure legends), or explain why this information doesn't apply to your submission:

eLife Sciences Publications, Ltd is a limited liability non-profit non-stock corporation incorporated in the State of Delaware, USA, with company number 5030732, and is registered in the UK with company number FC030576 and branch number BR015634 at the address 1st Floor, 24 Hills Road, Cambridge CB2 1JP | August 2014

Replicates were defined as follows (according to Blainey et al., Nature Methods, 11,879-880(2014))

One tissue sample: biological replicate
One individual mouse: biological replicate
Individual cortical slices: technical replicate
Individual cell: biological replicate
Individual dendritic segments: biological replicate
Individual dendritic spine: biological replicate

Statistics were made on biological replicates only.

All experiments have been replicated in at least 3 independent biological samples.

Figure Legends:
Figure 1: p. 34; Supplementary File 1
Figure 1-figure supplement 1: p. 35

Figure 2: pp. 36, 37
Figure 2-figure supplement 1: p. 38

Figure 3: p. 39
Figure 3-figure supplement 1: p. 40
Figure 3-figure supplement 2: p. 41

Figure 4: p. 42
Figure 4-figure supplement 1: pp. 43, 44

Figure 5: p. 45

Figure Response Letter: Response to the Decision Letter

Human tissue sample 5 has been used for anisomycin experiments only. (Supplementary File 1)
Human tissue sample 8 has been used for immunogold labeling experiments only (Supplementary File 1)

Inclusion/Exclusion-Criteria:
Defined inclusion criteria: Cortical slices from all human samples were macroscopically normal and showed no overt pathology. (p. 20; ll. 13, 14)

Exclusion criteria for electrophysiological recordings: Series resistance was monitored and recordings were discarded if series resistance reached >30 MΩ. (p. 22; ll. 12, 13)

Excluded data points (p. 22, ll. 14-23; p. 23, ll. 1-6):
One superficial (layer 2/3) cell in human neocortical slices (Figure 1; atRA group) with sEPSC amplitude = 38.4 pA and sEPSC frequency = 6.5 Hz showed interneuron characteristics and was therefore excluded from the analysis.

The series resistance of one cell from a human cortical slice (Figure 1; control group) exceeded 30 MΩ during I-V curve recording. The respective I-V-curve was therefore excluded from further analysis.

In five human superficial pyramidal neurons (Figure 1), the number of sweeps in the I-V-curve recordings was lower compared to other recordings (40 sweeps vs. 60 sweeps). Thus, cells were excluded from further analysis of action potential frequency.

Furthermore, one I-V-curve recording in the actinomycin-only treated group became unstable during the last sweeps and was consecutively excluded from action potential frequency analysis (Figure 4-figure supplement 1).

In addition, one cell from a mouse neocortical slice (Figure 3, Figure 3-figure supplement 1, and Figure Response Letter; *Synpo*$^{+/+}$, atRA group) was excluded from further analysis because the signal displayed a marked electrical interference that caused disturbances in the baseline of the recordings.

In the same data set, I-V-curve recording from one cell (Fig. S3, *Synpo*$^{+/+}$, control group) is excluded due to I-V duplication in each sweep.

Finally, one whole-cell patch-clamp recording of intrinsic cellular properties (Fig. S3; Thy1-GFP/Synpo, control group) lost its integrity during the recording and was therefore excluded from further analysis.

Data points outside the axis limits were reported in the figure legends.

Statistical reporting

- Statistical analysis methods should be described and justified
- Raw data should be presented in figures whenever informative to do so (typically when N per group is less than 10)
- For each experiment, you should identify the statistical tests used, exact values of N, definitions of center, methods of multiple test correction, and dispersion and precision measures (e.g., mean, median, SD, SEM, confidence intervals; and, for the major substantive results, a measure of effect size (e.g., Pearson's r, Cohen's d)
- Report exact p-values wherever possible alongside the summary statistics and 95% confidence intervals. These should be reported for all key questions and not only when the p-value is less than 0.05.

Please outline where this information can be found within the submission (e.g., sections or figure legends), or explain why this information doesn't apply to your submission:

Description of statistical tests (p. 27; ll. 1-14):
Statistical analyses were performed using the GraphPad Prism 7 software package. Two-group comparisons were performed using a Mann-Whitney-U test; U-values for statistically significant differences are reported in the figure legends. A Kruskal-Wallis test with Dunn's multiple comparisons was used to compare more than two groups. Correlation of individual data points was visualized by a linear regression fit and analyzed by computing Spearman r. For statistical evaluation of XY-plots from, we used a RM two-way ANOVA test (repeated measurements/analysis) with Sidak's multiple comparisons. For the comparison of more than two groups in XY-plots, Tukey's multiple comparisons were applied. For the in-sample analysis of human cortical slices (paired experimental design), we used a Wilcoxon matched-pairs signed-rank test. P values <0.05 were considered statistically significant (*$p < 0.05$, **$p < 0.01$, ***$p < 0.001$); results that did not yield significant differences are designated "ns". Statistical differences in XY-plots were indicated in the legend of the figure panels (*) when detected through multiple comparisons, irrespective of their localization and the level of significance. In the text and figures, values represent the mean ± standard error of the mean (s.e.m.).

All figure legends contain information as follows:
Figure 1 (p. 34): Individual data points are indicated by gray dots. Values represent mean ± s.e.m. (ns, non-significant difference, ** $p < 0.01$).

Figure 2 (pp. 36, 37): Individual data points are indicated by gray dots. Values represent mean ± s.e.m. (ns, non-significant difference, * $p < 0.05$, *** $p < 0.001$).

Figure 3 (p. 39): Individual data points are indicated by gray dots. Values represent mean ± s.e.m. (ns, non-significant difference, *** $p < 0.001$, ** $p < 0.01$).

Figure 4 (p. 42): Individual data points are indicated by gray dots. Values represent mean ± s.e.m. (ns, non-significant difference, * $p < 0.05$, ** $p < 0.01$).

Figure 5 (p. 45): Individual data points are indicated by gray dots. Values represent mean ± s.e.m. (ns, non-significant difference, *** $p < 0.001$).

Values of N were reported in the figure legends along with the respective statistical tests. (pp. 26-32; supplementary data)

(For large datasets, or papers with a very large number of statistical tests, you may upload a single table file with tests, Ns, etc., with reference to sections in the manuscript.)

Group allocation

- Indicate how samples were allocated into experimental groups (in the case of clinical studies, please specify allocation to treatment method); if randomization was used, please also state if restricted randomization was applied
- Indicate if masking was used during group allocation, data collection and/or data analysis

Please outline where this information can be found within the submission (e.g., sections or figure legends), or explain why this information doesn't apply to your submission:

Group Allocation:
Acute cortical slices prepared from one sample were randomly assigned to the respective treatment groups to (p. 21; ll. 7, 8).

Blinding:
If no automated analysis was applied, analysis was performed by an investigator blind to experimental conditions. (p. 26; ll. 4-22)

Additional data files ("source data")

- We encourage you to upload relevant additional data files, such as numerical data that are represented as a graph in a figure, or as a summary table
- Where provided, these should be in the most useful format, and they can be uploaded as "Source data" files linked to a main figure or table
- Include model definition files including the full list of parameters used
- Include code used for data analysis (e.g., R, MatLab)
- Avoid stating that data files are "available upon request"

Please indicate the figures or tables for which source data files have been provided:

GraphPad files for all figures are provided in a data repository for further evaluation.
https://datadryad.org/stash/share/4dLKdvPadcOyURdAKnehHijOzm-6QJpGHSLu1zpED00

Furthermore, we state that all original data are available upon reasonable request from the corresponding author (pp. 16; ll. 19, 20).
